# Whether the Research on Ethanol–Water Microstructure in Traditional Baijiu Should Be Strengthened?

**DOI:** 10.3390/molecules27238290

**Published:** 2022-11-28

**Authors:** Dan Qin, Yi Shen, Shiqi Yang, Guihu Zhang, Dongmei Wang, Hehe Li, Jinyuan Sun

**Affiliations:** 1 Key Laboratory of Brewing Molecular Engineering of China Light Industry, Beijing Technology and Business University, Beijing 100048, China; 2 College of Biosystems Engineering and Food Science, Zhejiang University, Hangzhou 310058, China; 3 Sichuan Langjiu Co., Ltd., Gulin 646523, China

**Keywords:** baijiu, alcoholic beverages, hydrogen bond, ethanol–water, flavor, interaction

## Abstract

Baijiu is a unique and traditional distilled liquor in China. Flavor plays a crucial rule in baijiu. Up to now, the research on the flavor of baijiu has progressed from the identification of volatile compounds to the research on key aroma compounds, but the release mechanism of these characteristic compounds is still unclear. Meanwhile, volatile compounds account for only a tiny fraction, whereas ethanol and water account for more than 98% of the content in baijiu. By summarizing the ethanol–water hydrogen bond structure in different alcoholic beverages, it was found that flavor compounds can affect the association strength of the ethanol–water hydrogen bond, and ethanol–water can also affect the interface distribution of flavor compounds. Therefore, the research on ethanol–water microstructure in baijiu is helpful to realize the simple visualization of adulteration detection, aging determination and flavor release mechanism analysis of baijiu, and further uncover the mystery of baijiu.

## 1. Introduction

**Figure 1:**
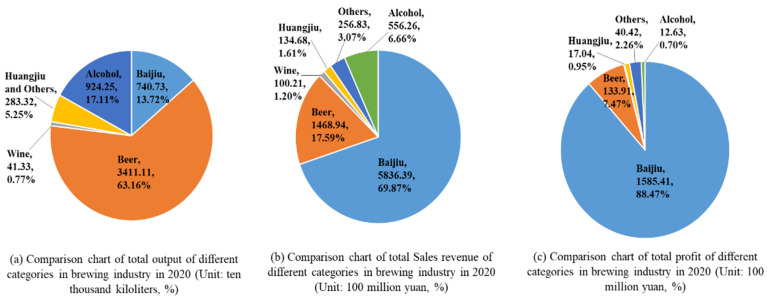
Comparison chart of total output (**a**), total sales revenue (**b**) and total profit (**c**) of different categories in brewing industry in 2020.

Baijiu is a unique and traditional distilled liquor in China with a history of more than 2000 years [1]. In 2021, the output of baijiu was 7.1563 billion liters (data from the China Alcoholic Drinks Association). The popularity of baijiu in China makes it the world’s most consumed spirit, and it has gained popularity overseas as well [2]. Meanwhile, the baijiu industry has developed into the main economic pillar in China’s brewing industry. According to the statistics of the China Alcoholic Drinks Association (Figure 1), the Baijiu industry achieved 69.87% of sales revenue and 88.47% of profits with a production capacity of 13.72% in 2020. The sales revenue of beer was only 25.17% of that of baijiu; huangjiu only 2.31% of that of baijiu; and wine, only 1.71% of that of baijiu. For a long time, baijiu has been an indispensable drink in celebrations, banquets, and daily life in China, and is widely regarded as China’s national liquor, and endowed with an important position in the Chinese food industry [3].

**Figure 2:**
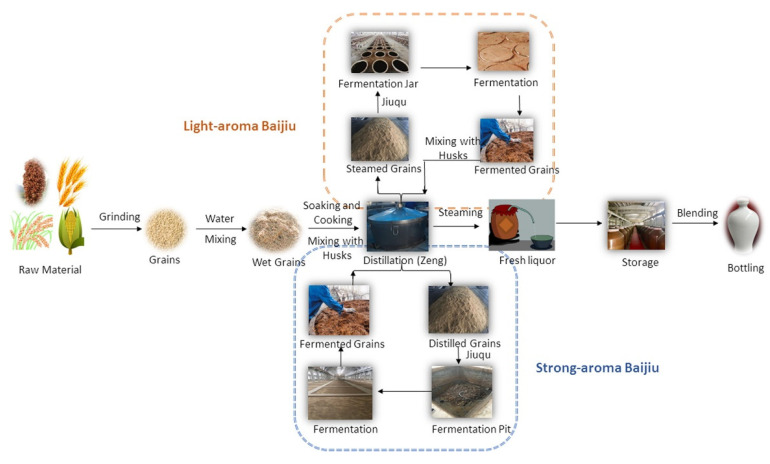
The traditional production processing of baijiu. Reprinted with permission from Ref. [1]. Copyright 2018, American Chemical Society.

**Figure 3:**
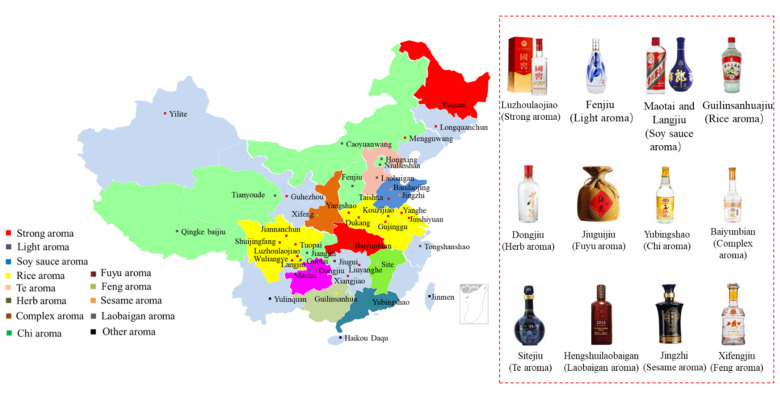
Distribution and representation of mainstream aroma-types of baijius in China.

Baijiu is one of the main six distilled spirits (baijiu, vodka, whiskey, brandy, rum and gin) in the world. Compared with other distilled spirits (brandy and whiskey), the fermentation of baijiu is a complex process involving saccharification and spontaneous fermentation simultaneously. Furthermore, baijiu is fermented and distilled under solid-state conditions [4]. The baijiu production process (as shown in Figure 2) mainly includes the following procedures: (i) raw materials (whole grains or powdered grains) are uniformly mixed; (ii) mixed grains are then fermented in a fermentation pit; (iii) fermented grains are distilled in Zen tanks to obtain the base baijiu (the alcohol content is usually above 50% vol.); (iv) base baijiu is then stored and aged in a pottery or stainless-steel vessel for several years; (v) finally, stored baijiu is blended to obtain products of different grades (alcohol content 38 to 65% vol.) [1]. The stored baijiu has usually been stored for 1~3 years. For instance, the storage period for the soy sauce aroma type of baijiu is more than 3 years, while that for light aroma type baijiu is about one year [1]. The diversity of brewing raw materials, brewing equipment, production technology, and geographical environment (temperature, humidity and altitude, etc.) determines the differences in the baijiu produced in different regions of the country. According to the aroma characteristics, baijiu can be classified into 12 categories [5,6], including the light, soy sauce, strong, sesame, rice, chixiang, texiang, complex, herb-like, laobaigan, fengxiang, and fuyu aroma types (as shown in Figure 3).

The flavor and taste highly influence the quality and acceptance of baijiu. Research has indicated the essential role of volatile compounds, including esters, alcohols, acids, aldehydes, ketones, nitrogen-containing and sulfur-containing compounds, etc., in the flavor of baijiu [1]. The volatile compounds account for only a tiny fraction, while ethanol and water take up more than 98% of the content of baijiu [7].

## 2. Research Advances on Flavor Chemistry of Baijiu

Flavor is an important indicator that determines the quality and consumer acceptance of baijiu. With the improvement in people’s living standards, their pursuit of baijiu has also increasing gradually, attracting more and more academic attention to the flavor of baijiu. Research on baijiu began in 1960, since the introduction of gas chromatography, and the main goal of baijiu flavor research was to identify the volatile compounds [4]. So far, more than 2020 volatile compounds, including esters, alcohols, acids, aldehydes, ketones, sulfur-containing compounds and nitrogen-containing compounds have been identified in baijiu [3]. However, in the above studies, not all the detected compounds have aroma or characteristic aroma, and their influence on the flavor of baijiu remains unknown. Since the research idea of “molecular sensory science” [8,9] was put forward, with the help of the research idea of flavor chemistry and sensory analysis as the benchmark, the second research focus has been placed on the key aroma compounds that affect the characteristic flavor of baijiu. With the development of gas chromatography–olfactometry, quantitative methods and flavor contribution studies, key aroma compounds are characterized in many different aroma-types baijius [10,11,12,13,14,15].

Given that volatile compounds alone are not enough for the overall flavor construction, further research focuses on the interaction of key compounds, and even volatile and non-volatile compounds. Zhu et al. [16] found that ethyl caproate, 2-methyl-3-furanthiol, 3-mercaptoacetate and 2-isopropyl-3-methoxypyrazine can all significantly lower the threshold of the aroma recombination model, mainly due to the synergistic effect of the four compounds with other volatile compounds in the baijiu sample. Yan et al. [17] studied the soy sauce aroma type of baijiu using the threshold method, and found that dimethyl sulfide, dimethyl disulfide and dimethyl trisulfide can effectively enhance the fruity flavor of baijiu. Wang et al. [18] claimed that, compared with the recombination model containing only aroma-active compounds, the odor of the recombination model involving the aroma-active compounds and the nonvolatile organic acids was more similar to that of the original baijiu sample.

There is a complex system in baijiu, composed of volatile compounds, nonvolatile compounds, ethanol, and water. Meanwhile, the interaction effect between different components is also complicated. The mixing of ethanol and water produces complex changes in macroscopic properties, which is closely related to the hydrogen bond association between ethanol and water to form new cluster molecules. In this case, in addition to the interaction of key compounds, coupled with the volatile and non-volatile compounds, there are also interactions between compounds and ethanol–water. However, only a few pieces of research have been carried out on the interaction between compounds with ethanol and water. Inspired by the microstructure of whiskies, tequilas and other beverages [19,20,21,22,23], studying the interaction between compounds and the hydrogen bond of ethanol–water and microscopic images is conducive to further investigating the flavor of baijiu and uncovering the mystery of baijiu.

## 3. Hydrogen Bonding Structure of Ethanol and Water in Solution Chemistry

The hydrogen atom (H) is covalently bonded to atom X (F, O, N, etc.) with a smaller radius and greater electronegativity. If covalently bonded to the atom Y with a greater electronegativity (or the same as X), hydrogen can be used as the medium between X and Y to produce a particular intramolecular or intermolecular interaction in the form of X-H---Y, i.e., the hydrogen bond [24]. The hydrogen bond is slightly stronger than van der Waals forces, but weaker than the ionic or covalent bonds. The mixing of ethanol and water produces complex changes in macroscopic properties, which is closely related to the hydrogen bond association between ethanol and water to form new cluster molecules. Regarding the research on the molecular structure of water, ethanol and their aqueous solutions, a series of approaches including nuclear magnetic resonance spectroscopy (NMR), Raman spectroscopy, infrared spectroscopy, microwave, X-ray diffraction, neutron diffraction, ab initio algorithm, molecular dynamics (MD) simulation, etc., were all thereby adopted to study the structure of hydrogen bonds.

NMR is a time-efficient, reproducible and non-destructive powerful tool, and does not require laborious sample preparation [25]. The intermolecular hydrogen bond combination is described by ^1^H-NMR in detail, and reflects the conditions of the three hydrogen bond interactions, i.e., ethanol–water, ethanol–ethanol, and water–water. Fluorescence spectroscopy is a fast, sensitive and non-destructive analysis technique that provides information about fluorescent molecules [26,27]. The probes of dynamic light scattering (DLS) and small-angle X-ray scattering (SAXS) can cover a wide size range of structures, with DLS 10 to 1000 nm and SAXS sub-nm to 100 nm. The size and concentration of mesoscopic systems can also be determined by DLS and SAXS [28]. Atomic Force Microscopy (AFM) functions based on the interaction between the probe and the sample, and can recognize three-dimensional imaging with nanometer resolution, especially in the vertical direction [29]. MD simulation is a computer simulation method that investigates the physical movements of atoms and molecules, using the interaction potential between atoms or the force field of molecular mechanics and making it possible to track and understand the structure and dynamics during the training of individual atoms [30].

**Figure 4:**
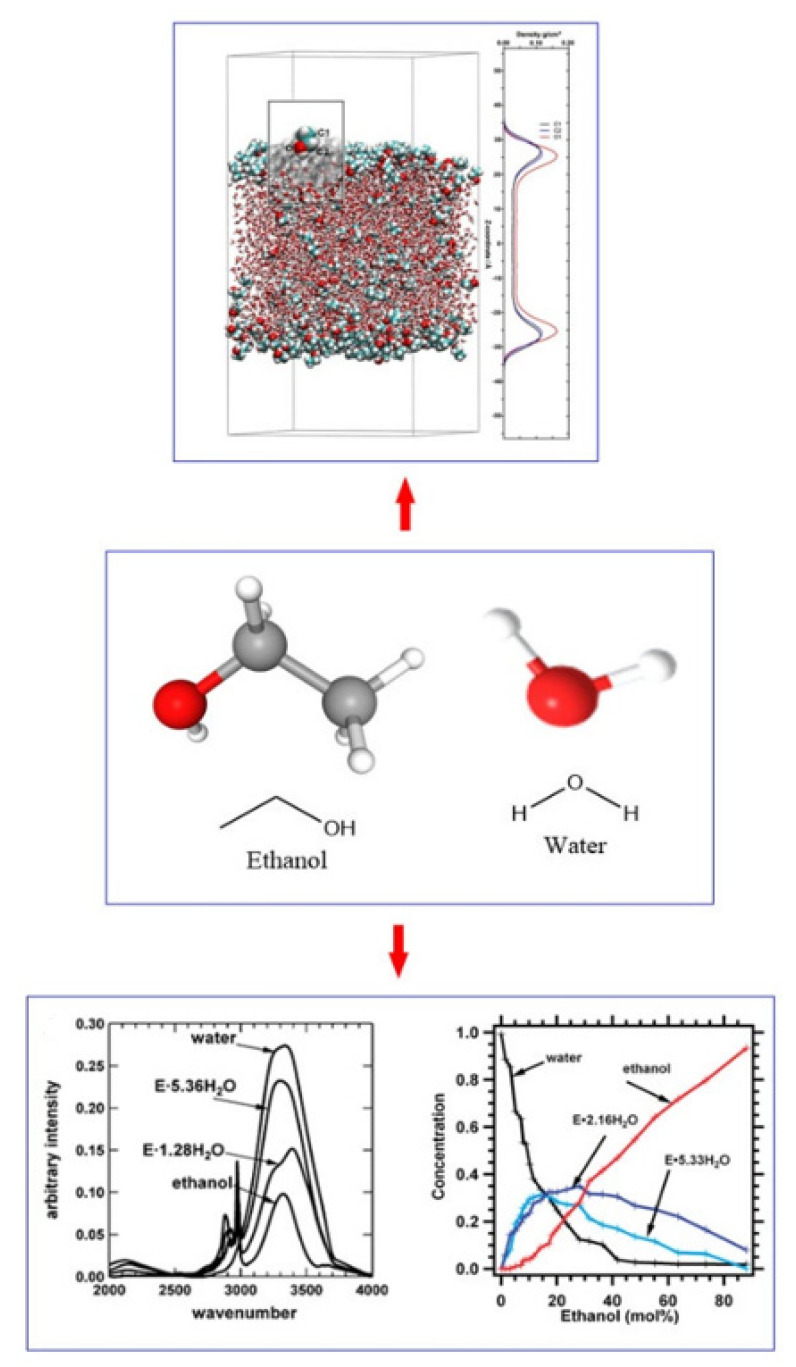
The hydrogen bond structure characterization of ethanol–water.Reprinted with permission from Ref. [31]. Copyright 2010, American Chemical Society; Reprinted with permission from Ref. [32]. Copyright 2017, The Author(s).

Hydrogen bonds of ethanol and water were studied earlier in solution chemistry and had aroused increasing concern. Compared with the corresponding pure components, ethanol–water mixtures often present different properties compared with the corresponding pure components. The structure and diffusion properties matter considerably in theoretical research and technical application. The hydrogen bond structure of ethanol–water is usually characterized by instrumental studies and molecular dynamics simulations, as shown in Figure 4.

There are three interaction terms of hydrogen bonds, i.e., ethanol–ethanol, ethanol–water, and water–water during the ethanol–water solutions. Ratajska-Gadomska showed that the Raman spectrum of O-H stretching vibration in aqueous ethanol solution reflects the number of hydrogen bonds among solute–solute, solute–solvent and solvent–solvent molecules determined by the ethanol concentration [33]. The strength of the H-bonding was evaluated using the intensity ratio of the OH stretch bands at 3200 and 3420 cm^−1^ for Raman spectra and 3240 and 3360 cm^−1^ for IR spectra. According to the ^1^H NMR experiments, hydro–alcoholic solutions exhibit extreme behaviors at 25%, 46% and 83% vol. The application of near-infrared spectroscopy confirms the presence of four important compounds (“individual” ethanol and water structures, and two defined water–ethanol complexes 1:1 and 1:3) [34]. H-bonding strength in the water–ethanol mixed solution is the largest in an ethanol concentration of 15–20% *w*/*w* [35]. Li et al. [36] found that with the increase of ethanol concentration, the symmetric and asymmetric OH stretching vibrational mode (3286 and 3434 cm^−1^) of water shifts to a lower frequency, and the weak shoulder at 3615 cm^−1^ disappears. The results show that ethanol strengthens hydrogen bonds in water. As for the ethanol–water correlation, the strong interaction between ethanol and water increases the ethanol–water structure with ethanol concentration [37]. Gong found that mixing alcohol and water can soften and extend the H-O bond and O: H non-bond of the water–HB network [38]. 

The mid-infrared spectrum of the ethanol–water solution was analyzed using the multivariate curve resolution-alternating least square method (MCR-ALS), which explains the structural basis of the NMR spectrum pattern change. The results show that the difference in the NMR spectrum is attributed to the ethanol–ethanol clusters [39]. Two-dimensional correlation analysis reveals that in the concentration of 10–40%, ethanol first interacts with water molecules, but the self-association between ethanol molecules is preferred in the concentration of 40 to 100%, which weakens the interaction between water and ethanol, and disintegrates water and ethanol clusters [40]. The concentration dependences of the self-diffusion coefficients of ethanol and water molecules in ethanol water solutions were obtained by NMR with the pulsed magnetic field gradient [41]. According to the time-resolved spectrum, the possible bonding constant n of the molecular cluster is calculated to be two, and the molecular cluster is concluded as a hydrogen bond chain structure formed by an ethanol molecule and two water molecules [42].

Asenbaum et al. [43] studied the structural changes in ethanol–water mixtures by ultrasonics, brillouin scattering and MD simulation, and found the major structural rearrangements within the composition range of 0.15 < x_EtOH_ < 0.20 for the liquid system (x_EtOH_C_2_H_5_OH + (1 − x_EtOH_)H_2_O) under ambient temperatures and pressures. In addition, a corresponding change in the structure–vibration dynamics of the water and ethanol domains forms the bicontinuous mixture in 0.15 < x_EtOH_ < 0.65. Chain-like hydrogen-bond clusters of water are observed at larger ethanol concentrations. The composition analysis of FT-IR and Raman spectroscopy presents a kind of water-rich hydrate of E^.^(5.3 ± 0.1)H_2_O in both vodka and water–ethanol solutions [31]. The fluorescence spectrum has shown that the main cluster is (H_2_O)_m_(EtOH) in the low concentration range of 10–45%, which corresponds to the peak at 373 nm, while in the 80–100% concentration range the main cluster is (H_2_O)(EtOH)_n_, corresponding to the emission peak at 308 nm. Meanwhile, the most stable (H_2_O)_m_(EtOH)_n_ cluster, corresponding to the peak at 330 nm, not only predominately accounts for the concentration range of 50–75%, but also presents in all mixtures during a long incubation time [44]. Huang et al. [45] researched the structural stability of the binary and ternary complexes by density functional theory, and found that the structural stability is in the decreasing order of ethanol/water–acid > ethanol/water–alcohol > ethanol/water–ester, and ethanol–water–acid > ethanol–water–alcohol > ethanol–water–ester, respectively.

## 4. Hydrogen Bonding Structure of Ethanol and Water in Alcoholic Beverages

In terms of the brewing science, the research on hydrogen bond properties of ethanol–water has not received as much attention as solution chemistry. The present research aims to provide guidance for future research on the hydrogen bond structure of ethanol and water in baijiu by reviewing the hydrogen bond structure of ethanol and water in baijiu and other beverages.

### 4.1. Baijiu

Gu, E. et al. [46] researched the three-dimensional fluorescence spectra and absorption of Hai zhilan, Tian zhilan and Meng zhilan using a UV-240 ultra-violet spectrophotometer and an Sp-2558 multifunctional spectrometer. The results of three-dimensional fluorescence spectroscopy show that each baijiu emits three strong fluorescence lines at 310, 420, and 610 nm, respectively. Under the ultraviolet excitation at 245 nm, the fluorescence peaks reflect the aging process and different contents of baijiu. Qiao, H. et al. [47] investigated the ethanol–water association behavior and total hydrogen bond properties of Fenjiu using fluorescence and viscosity measurement. The results show that the ethanol–water hydrogen bond strength and total hydrogen bond strength of aged Fenjiu are heavily dependent on the total ester lost and the sodium ion obtained in the ceramic container, not only on the aging time. Gao, B. [48] found that temperature is the main factor affecting the strength of hydrogen bonds of ethanol–water in Fenjiu. Furthermore, given that ethanol is released and exposed to the medium with the ethanol–water system disintegrating, and causes a spicy irritation, the taste of 60% of baijiu decreases rapidly with the increase of the storage temperature. Huang et al. [45] found that organic acids with ethanol or water could form ethanol/water–acid and ethanol–water–acid ring hydrogen bond structures in baijiu. This may be the reason that baijiu showed an increase in acids and a decrease in esters during baijiu aging.

### 4.2. Whiskey

To date, there have been thousands of compounds. Whiskey is produced by the fermentation of grain sources, distillation of alcohol, and its maturation, and its production involves several key stages. Malt whiskey is made from malted barley, while grain whiskey is made from malted barley and other grains [49]. Many whiskies, especially those brewed on the Scottish island of Islay, convey a typical smoky flavor, which is from smoking the malt over a peat fire. Whiskey usually goes through a long-term maturation process in wooden barrels or casks, and the reduction in the pungent taste after maturing may have a close association with water and ethanol molecules [50].

Nose, A. et al. [51] investigated the hydrogen bond structure of ethanol–water in whiskey (including 32 malt whiskies, aged for 0–23 years) by ^1^H NMR on the OH chemical shift of water and ethanol, and found that, in addition to the aging time, the hydrogen bond strength of aging whiskey is also related to the acidity and phenolic compounds in oak barrels. Karlsson, B.C.G. et al. [32] explained that diluting whiskey with water affects its taste, and found that guaiacol is related to ethanol–water in whiskey. To clarify the relationship between the liquid structure of whiskey and flavor maturity, Morishima, K. et al. [28] measured the mesoscopic structure using dynamic light scattering and small-angle X-ray scattering. The results show that the small cluster component is the key to obtaining flavored whiskey, while the concentration of large cluster components is related to the alcohol irritation of whiskey, having nothing to do with the maturity time.

### 4.3. Vodka

Vodka is a tasteless and colorless alcoholic beverage fermented and distilled from grains, potatoes, beets, grapes or cassava [52]. In vodka production, the alcohol obtained from the fermentation and distillation processes undergoes further processing, such as charcoal or carbon filters. Then, rectified spirit and desalted water in correct ratios are mixed, and additional filtration is performed before bottling to obtain the final product [53].

Hu, N. et al. [31] studied the difference in hydrogen bond strength between vodka using ^1^H NMR, FT-IR, and Raman spectroscopy. The component analysis of FT-IR and Raman data presents a water-rich hydrate of E^.^(5.3 ± 0.1)H_2_O in both vodka and water-ethanol solutions. The study demonstrates the correlation of the hydrate structure E^.^(5.3 ± 0.1)H_2_O and its content with the perception of vodka.

### 4.4. Japanese Sake

Japanese sake is made from rice and water through a unique process, involving two kinds of microorganisms, fungus and yeast. Rice is the primary raw material for sake brewing, and the characteristic of the rice is one of the critical factors that determine the quality of the final product. In sake brewing, steamed rice has two types of application: i.e., to be added directly to sake mash, and to make koji. The taste and aroma of sake are related to many components [54]. 

Nose, A. et al. [51] demonstrated that the strength of Japanese sake’s ethanol–water hydrogen bond is associated with the total concentration of organic acids and amino acids that cause low-field chemical shifts. The results also show that compounds originating from the raw material, rice, or other products affect the hydrogen-bonding structure in Japanese sake. In shochu, the presence of a small amount of organic acid can strengthen the hydrogen bond structure of water–ethanol and promote the proton exchange between water–ethanol molecules at the same time. In fruit cocktails, the hydrogen bonding of water–ethanol is strengthened by organic acids and (poly)phenols from fruit juices [55].

### 4.5. Fermented Alcoholic Beverages

Fermented alcoholic beverages refer to beverages produced by fermentation or partial fermentation, with grains, fruits, and milk as the primary raw materials. Huangjiu, a traditional Chinese fermented alcoholic beverage, is also an ethanol and water solution featuring a unique flavor and taste, acknowledged as one of the most popular alcoholic beverages in China with an annual consumption of millions of liters [56]. 

Cao, J. et al. [57] investigated the structure of ethanol–water, defined the strength of hydrogen bonding by ^1^H-NMR and viscosity measurement and studied the relationship between hydrogen bond strength and flavor in Chinese rice wine. The results provide an essential theoretical basis for the molecular mechanism and quality control of increased viscosity during rice wine aging. Chiappisi, L. et al. [58] probed the microstructure of limoncello using small-angle neutron scattering, and revealed the existence of 100 nm droplets spontaneously formed in a large composition and temperature range. The results are not limited to limoncello but can also be extended to rapidly evolving formulas based on water-insoluble oils, water, and alcohols.

## 5. Interactions between Flavor Compounds and Hydrogen Bonding of Ethanol and Water in Beverages

Flavor plays a key role in beverages. Due to the differences in brewing raw materials, production technology and storage conditions, etc., different alcoholic beverages such as baijiu, whiskey, brandy and wine, etc., have their own unique flavor, and the compounds in these beverages are also different. For example, ethyl hexanoate (pineapple, 29–59,594), ethyl octanoate (fruity, OAV, 782–9443), ethyl butanoate (fruity, 22–9051), ethyl acetate (fruity, 12–81), 3-methylbutanol (green, 2–3), acetic acid (vinegar, 2–7), hexanoic acid (sweaty, 7–291), phenylacetaldehyde (floral, 5–24), etc., were significant flavor compounds in baijiu due to their high OAV value (OAV > 1). Hexanol (floral, OAV 41,000), E) -β-damascenone (floral, honey, 1800) and ethyl (S)-2-methylbutanoate (sweet, 1700), etc., were significant flavor compounds in brandy. Ethyl (S)-2-methylbutanoate (sweet, 138), 3-methylbutanal (malt, 122), etc., were significant flavor compounds in whiskey. (E)-β-damascenone (floral, honey, OAV 1100), ethyl butanoate (fruity, 442), ethyl hexanoate (pineapple, 145), etc., were significant flavor compounds in rum [59]. Considerable research has indicated the effect of some compounds originating from raw materials, rice, or products produced by the microorganisms during the fermentation of ethanol on the hydrogen bond structure of Japanese sake [50]. Studies have also confirmed the efficiency of the chemical components in alcoholic beverages (such as whiskey, Japanese sake, shochu) in strengthening the hydrogen bond structure [60]. Compounds (such as ethyl acetate, acetaldehyde, methanol and fusel oils, etc.) can impact the molecular water restructuring by MD simulation and equilibrium radial distribution function (RDF) analysis of simulated aqueous ethanol solution [61]. OW–OW’s peak height increases linearly with the number of carbon atoms in the compound until the number of carbon atoms exceeds four. When the number of carbon atoms is greater than ethanol molecule (C > 2), the peak heights of OE–OW and OE–OE increase correspondingly.

In the ethanol concentration of 20% *v*/*v*, the OH peaks of water and ethanol are merged into a single peak in the ^1^H NMR spectrum. Acids (H+ and HA: undissociated acids) and bases (OH– and A–: conjugate-base anions from weak acids) can strengthen the structure of the water–ethanol hydrogen bond [60]. The conclusion drawn by Cao et al. [57] is consistent with the results by Nose, A. In addition, citric acid, malic acid, benzaldehyde, ethyl lactate, isoamyl alcohol, 2-phenylethyl and ethyl acetate are also considered factors affecting the strength of hydrogen bond from strong to weak. 

The effect factors are shown in Table 1. Sugars, most acids, alcohols, esters and phenols can cause OH proton chemical shifts to move to low fields, thereby increasing the hydrogen bond association between ethanol and water. However, aldehydes can cause OH proton chemical shifts to move to high fields, and thus decrease the strength of the hydrogen bond association between ethanol and water. Additionally, MgCl_2_ and MgSO_4_ can increase while other salts decrease the strength of the hydrogen bond association between ethanol and water. In addition, some compounds such as maltose, glucose, ethyl acetate, ethyl lactate and NaCl, etc., have almost the same concentration ranges but the strength is completely or partly opposite; the difference may be caused by the different ratio of ethanol/water or the experimental apparatus random error caused by the different experimental conditions. The effect of acetic acid, which has almost the same concentration range, on the strength of hydrogen bond association was consistent at different ratios of ethanol/water of 18% *v*/*v*, 20% *v*/*v*, 25% *v*/*v* and 60% *v*/*v*. With an increase in acetic acid concentration, the hydrogen bond association strength of ethanol–water increases gradually. With an increase in MgCl_2_ concentration, the hydrogen bond association strength of ethanol–water increases gradually no matter the ratio of ethanol/water [47,55,62,63].

**Figure 5:**
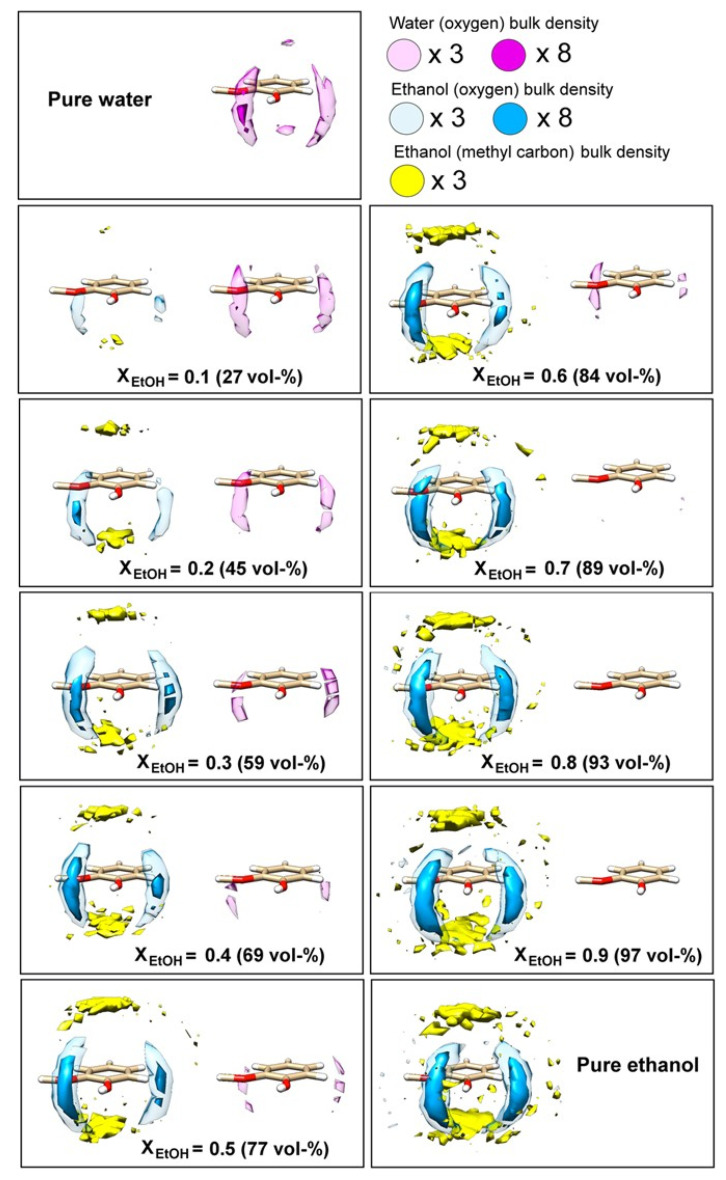
Solvent affinity of water and EtOH for guaiacol. Atomic spatial distribution functions (SDFs) of water oxygen and EtOH oxygen and methyl carbon components around a single guaiacol molecule in liquid–air interface mixtures of varying EtOH content. Reprinted with permission from Ref. [32]. Copyright 2017, The Author(s).

Ickes, C.M. et al. [64] reviewed the effect of ethanol on the flavor perception of alcoholic beverages, and found that the physical and chemical properties of ethanol–water mixtures matter considerably in understanding the complex effects of ethanol on the flavor perception and release of alcoholic beverages. For example, the lower ethanol concentration in wines had a higher fruity flavor, and increasing the ethanol concentration decreases the worty flavor in beer. The degree of physiological inhibition of the aroma by ethanol is not universal, and some compounds are more affected than others, indicating the existence of a potential relationship between the inhibition of aroma by ethanol and the structure of aromatic molecules [65]. Karlsson et al. [32] performed a series of spatial distribution functions (SDFs) and found the probabilities of water and ethanol around the guaiacol in a 50 ns liquid–air interface simulation. The probability of the numerator is shown in Figure 5. Both ethanol and water oxygen atoms are more inclined to the phenol ring of guaiacol. In this position, the hydroxyl groups of water and ethanol can interact with any group through hydrogen bonds. Such a hydrogen bonding pattern seems to significantly influence the structure of the first solvation shell, as shown in Figure 5. Above and below the guaiacol benzene ring is preferentially the methyl group of EtOH, revealing a stacking interaction between the ethyl group of ethanol and the guaiacol benzene ring in all ethanol–ethanol mixtures. In conclusion, flavor compounds can affect the association strength of ethanol–water hydrogen bonds, and ethanol and water can also affect the interface distribution of flavor compounds.

## 6. Discussion

Baijiu is a complex system composed of more than 2000 volatile compounds, non-volatile compounds, ethanol, water, etc. Flavor plays a crucial role in baijiu. Up to now, research on the flavor of baijiu has progressed from the identification of volatile compounds to the study of characteristic aroma compounds or even the interaction between the characteristic compounds, but the release mechanism of these characteristic compounds in baijiu is still unclear. Indeed, volatile compounds are necessarily important in the flavor of baijiu, but account for only a tiny fraction, while ethanol and water take up more than 98% of the content of baijiu. Ethanol and water can also affect the interface distribution of flavor compounds. However, the interaction between volatile compounds and ethanol–water is rarely studied. Through the study of the ethanol–water hydrogen bond structure, the interaction between ethanol–water and flavor compounds and the microstructure of baijiu, it is helpful to realize the simple visualization of adulteration detection, aging determination and flavor analysis of baijiu, and further uncover the mystery of baijiu.

## Figures and Tables

**Table 1:** Effect factors of hydrogen bonding strength in different ethanol–water systems (From Refs. [47,50,51,55,57,62,63]).

	Compounds	Concentration Ranges /mol/L	Hydrogen Bonding Strength	Ethanol–Water (*v*/*v*)/%	Instruments	Ref
**Sugars**	maltose	1 × 10^−5^~1	increase	18%	^1^H-NMR	[57]
		0~1	decrease	20%	^1^H-NMR	[51]
	glucose	1 × 10^−5^~1	increase	18%	^1^H-NMR	[57]
		0~1	decrease	20%	^1^H-NMR	[51]
	γ-cyclodextrin	0~1	decrease	20%	^1^H-NMR	[51]
	D-sorbitol	0~1	no change	20%	^1^H-NMR	[51]
**Acids**	citric acid	1 × 10^−5^~1	increase	18%	^1^H-NMR	[57]
		0~4		20%	^1^H-NMR	[63]
	malic acid	1 × 10^−5^~1	increase	18%	^1^H-NMR	[57]
		0~4		20%	^1^H-NMR	[63]
	pyruvic acid	1 × 10^−5^~1	increase	18%	^1^H-NMR	[57]
	formic	0~1	increase	20%	^1^H-NMR	[51]
	acetic acid	1 × 10^−5^~1	increase	18%	^1^H-NMR	[57]
		0~4		20%	^1^H-NMR	[63]
		1 × 10^−5^~1 × 10^−1^		25%	^1^H-NMR	[55]
		1 × 10^−7^~1 × 10^−1^		60%	^1^H-NMR	[63]
		1 × 10^−6^~1	first increase and then decrease	60%	Fluorescence spectroscopy	[47]
	oxalic acids	0~1	increase	20%	^1^H-NMR	[51]
	L-(+)-ascorbic acid	0~1	increase	20%	^1^H-NMR	[51]
	(+)-catechin	0~1	increase	20%	^1^H-NMR	[51]
	caffeine	0~1	decrease	20%	^1^H-NMR	[51]
	vanillic acid	1 × 10^−5^~1 × 10^−1^	increase	25%	^1^H-NMR	[55]
	lactic acid	1 × 10^−5^~1	increase	18%	^1^H-NMR	[57]
		0~4		20%	^1^H-NMR	[63]
	succinic acid	0~4	increase	20%	^1^H-NMR	[63]
	tartaric acid	0~4	increase	20%	^1^H-NMR	[63]
	phosphoric acid	0~4	increase	20%	^1^H-NMR	[63]
	trifluoroacetic acid	0~4	increase	20%	^1^H-NMR	[63]
	methanesulfonic acid	0~4	increase	20%	^1^H-NMR	[63]
	pyrogallol	0~1	increase	20%	^1^H-NMR	[63]
		1 × 10^−7^~1 × 10^−1^	increase	60%	^1^H-NMR	[63]
	benzoic acid	0~1	increase	20%	^1^H-NMR	[63]
		1 × 10^−7^~1 × 10^−1^	increase	60%	^1^H-NMR	[63]
	gallic acid	0~1	increase	20%	^1^H-NMR	[63]
		1 × 10^−7^~1 × 10^−1^	increase	60%	^1^H-NMR	[63]
	tannic acid	0~1	increase	20%	^1^H-NMR	[63]
	chlorogenic acid	0~1	increase	20%	^1^H-NMR	[63]
**Alcohols**	phenylethyl alcohol	1 × 10^−5^~1	increase	18%	^1^H-NMR	[57]
	isoamyl alcohol	1 × 10^−5^~1	increase	18%	^1^H-NMR	[57]
		1 × 10^−6^~1	first decrease and then increase	60%	Fluorescence spectroscopy	[47]
**Esters**	ethyl acetate	1 × 10^−5^~1	increase	18%	^1^H-NMR	[57]
		1 × 10^−6^~1	first increase and then decrease	60%	Fluorescence spectroscopy	[47]
	ethyl lactate	1 × 10^−5^~1	increase	18%	^1^H-NMR	[57]
		1 × 10^−6^~1	first increase and then decrease	60%	Fluorescence spectroscopy	[47]
**Aldehydes**	acetaldehyde	1 × 10^−5^~1	decrease	18%	^1^H-NMR	[57]
		0~1	decrease	60%	^1^H-NMR	[51]
	4-methyl benzaldehyde	1 × 10^−5^~1	decrease	18%	^1^H-NMR	[57]
	benzaldehyde	0~1	increase	60%	^1^H-NMR	[51]
	p-hydroxybenzaldehyde	0~1	increase	60%	^1^H-NMR	[51]
**Phenols**	phenol	0~1	increase	20%	^1^H-NMR	[63]
	epigallocatechin gallate	0~1	increase	20%	^1^H-NMR	[63]
	vanillin	1 × 10^−5^~1 × 10^−1^	increase	25%	^1^H-NMR	[55]
**Lactones**	D-(−)-pantolactone	0~1	first increase and then decrease	60%	^1^H-NMR	[51]
	D-(−)-pantolactone	0~1	decrease	20%	^1^H-NMR	[51]
	γ-octanolactone	0~1	first increase and then decrease	60%	^1^H-NMR	[51]
**Salts**	MgCl_2_	0~1	increase	3%	^1^H-NMR	[63]
		0~0.6		15%	^1^H-NMR	[62]
		0~8		20%	^1^H-NMR	[63]
		1 × 10^−5^~1		40%	^1^H-NMR	[62]
		0~1		60%	^1^H-NMR	[63]
	KF	0~8	increase	20%	^1^H-NMR	[63]
	LiCl	0~8	decrease	20%	^1^H-NMR	[63]
	NaCl	0~1	decrease	3%	^1^H-NMR	[63]
		0~0.6		15%	^1^H-NMR	[62]
		0~8		20%	^1^H-NMR	[63]
		1 × 10^−5^~1		40%	^1^H-NMR	[62]
		0~1		60%	^1^H-NMR	[63]
		1 × 10^−7^~1 × 10^−1^	first increase and then decrease	25%	^1^H-NMR	[63]
		1 × 10^−6^~1	increase	60%	Fluorescence spectroscopy	[47]
	NaHCO_3_	0~0.6	increase	15%	^1^H-NMR	[62]
		1 × 10^−5^~1		40%	^1^H-NMR	[62]
	Na_2_SO_4_	0~0.6	decrease	15%	^1^H-NMR	[62]
		1 × 10^−5^~1	first increase and then decrease	40%	^1^H-NMR	[62]
	CaCl_2_	0~0.6	decrease	15%	^1^H-NMR	[62]
		0~8		20%	^1^H-NMR	[63]
		1 × 10^−5^~1		40%	^1^H-NMR	[62]
	KCl	0~0.6	decrease	15%	^1^H-NMR	[62]
		0~8		20%	^1^H-NMR	[63]
		1 × 10^−5^~1	first increase and then decrease	40%	^1^H-NMR	[62]
	MgSO_4_	0~0.6	increase	15%	^1^H-NMR	[62]
		1 × 10^−5^~1		40%	^1^H-NMR	[62]
	RbCl	0~8	decrease	20%	^1^H-NMR	[63]
	CsCl	0~8	decrease	20%	^1^H-NMR	[63]
	NH4Cl	0~8	decrease	20%	^1^H-NMR	[63]
	LiBr	0~8	decrease	20%	^1^H-NMR	[63]
	NaBr	0~8	decrease	20%	^1^H-NMR	[63]
	LiI	0~8	decrease	20%	^1^H-NMR	[63]
	NaI	0~8	decrease	20%	^1^H-NMR	[63]
	NaOH	1 × 10^−7^~1 × 10^−1^	increase	60%	^1^H-NMR	[63]
	NaH_2_A	0~1.5	increase	20%	^1^H-NMR	[50]
	Na_2_HA	0~1.5	increase	20%	^1^H-NMR	[50]
	Na_3_A	0~1.5	decrease	20%	^1^H-NMR	[50]
	NaNO_3_	0~1	decrease	20%	^1^H-NMR	[51]
	NaHCO	0~1	no change	20%	^1^H-NMR	[51]
	sodium acetate	0~1	no change	20%	^1^H-NMR	[51]
**Amino acids**	glycine	0~1.5	increase	20%	^1^H-NMR	[50]
	DL-α-alanine	0~1.5	increase	20%	^1^H-NMR	[50]
	L(+)-arginine	0~1.5	increase	20%	^1^H-NMR	[50]
	L(+)-glutamine	0~1.5	increase	20%	^1^H-NMR	[50]
	L(-)-proline	0~1.5	increase	20%	^1^H-NMR	[50]
	L(+)-glutamate	0~1.5	increase	20%	^1^H-NMR	[50]
**Amines**	diethylamine	0~1.5	no change	20%	^1^H-NMR	[50]
	monoethanolamine	0~1.5	decrease	20%	^1^H-NMR	[50]
	propylamine	0~1.5	decrease	20%	^1^H-NMR	[50]

## Data Availability

Not applicable.
